# Positive Emotions at Work and Job Crafting: Results From Two Prospective Studies

**DOI:** 10.3389/fpsyg.2019.02786

**Published:** 2019-12-20

**Authors:** Anna Rogala, Roman Cieslak

**Affiliations:** ^1^Faculty of Psychology, SWPS University of Social Sciences and Humanities, Warsaw, Poland; ^2^Trauma, Health, and Hazards Center, University of Colorado, Colorado Springs, CO, United States

**Keywords:** job crafting, positive emotions, collective flow, self-efficacy, broaden-and-build theory, social cognitive theory

## Abstract

To date, research confirmed the effects of job crafting on the functioning of employees and organizations. In contrast, the evidence for the predictors of job crafting is limited. Based on broaden-and-build (B&B) theory, it may be assumed that high positive emotions at work would predict high job crafting behaviors at follow-ups. In line with social cognitive theory (SCT), it may be hypothesized that self-efficacy would mediate the relationship between positive emotions at work and following job crafting behaviors. The hypotheses were tested in a three-wave prospective study (Study 1, *N* = 124), with individual beliefs measured as the predictors. In a three-wave prospective Study 2 (*N* = 99), individual perceptions of collective flow at work and collective efficacy were assessed. Results of Studies 1 and 2 indicated that positive emotions at work predicted increasing structural resources, a job crafting dimension. Moreover, findings of Study 2 showed that collective flow at work predicted another job crafting dimension, i.e., increasing social resources. These results may inform good practices and help in designing individual- and team-level interventions enhancing job crafting behaviors.

## Introduction

Job redesign is usually perceived as driven by supervisors or an organization process leading to changing some characteristics of a job. However, there is also a different perspective on this process—job redesign may be initiated and performed by an employee without or with limited engagement of organizational resources. In this view, employees “customize their jobs to their individual needs and preferences, instead of reactively performing the job that the organization created” (Tims and Bakker, 2010, p. 1). These proactive behaviors depending on the individual’s initiative are called *job crafting* (Wrzesniewski and Dutton, 2001; Tims and Bakker, 2010). Positive consequences of job crafting for both individuals and their workplaces are widely researched and recognized (see Rudolph et al., 2017). Therefore, it is important to identify antecedents and mechanisms responsible for job crafting development.

Building on broaden-and-build (B&B) theory (Fredrickson, 2001), we suggest that positive emotions at work predict job crafting behaviors. Moreover, integrating the B&B theory (Fredrickson, 2001) and social cognitive theory (SCT; Bandura, 2001), we argue that self-efficacy mediates the relationship between positive emotions and job crafting behaviors. In Study 1, we conceptualize all variables at the individual level. To include teamwork context in Study 2, we measure both independent variable and mediator at the collective level, assessing individual perceptions of collective flow and collective efficacy. In Study 1, we verify the predictive role of positive emotions at work. In order to gain more knowledge about job crafting antecedents, in Study 2, we examine the role of collective flow at work as a predictor of job crafting.

### Job Crafting

Wrzesniewski and Dutton (2001) characterized job crafting as “the physical and cognitive changes individuals make in the task or relational boundaries of their work” (p. 179). This definition limits job crafting to three forms—changes made by employees in their job tasks, job relationships, and the meaning of the job. Recent research suggests that employees may also alter other aspects of their jobs, e.g., through personal skill development (Lyons, 2008; Tims et al., 2012; Petrou et al., 2015). To cover a broader scope of job characteristics that employees can craft, Tims and Bakker (2010) suggested framing job crafting within the job-demands resources (JD-R) model.

The JD-R model classifies all job aspects into two broad categories: job demands and job resources. Job demands involve those job characteristics that “require sustained physical and/or psychological (cognitive and emotional) effort or skills” (Bakker and Demerouti, 2007, p. 312), e.g., organizational constraints or workload. Job resources include those job characteristics that “are either/or functional in achieving work goals, reduce job demands and the associated physiological and psychological costs, and stimulate personal growth, learning, and development” (Bakker and Demerouti, 2007, p. 312), e.g., autonomy or social support. According to the JD-R model, “chronic job demands (e.g., work overload, emotional demands) exhaust employees’ mental and physical resources and may therefore lead to the depletion of energy (i.e., a state of exhaustion) and to health problems” (Bakker and Demerouti, 2007, s. 313). Therefore, employees tend to decrease their job demands when these exceed their capabilities (Bakker and Demerouti, 2007). Although not all of job demands are related to negative work outcomes, according to LePine et al. (2005), job demands can be categorized as either challenging job demands or hindering job demands. The former refers to job demands connected with goal achievement and personal growth of the employee when the latter refers to job demands that interfere with the employee’s ability to attain valued goals. Because challenging job demands produce positive work outcomes employees tend to increase them. They also tend to increase job resources, because of their role in initiating motivational process and therefore predicting positive work outcomes (e.g., work engagement). Moreover, job resources may buffer the effect of job demands on negative work outcomes (e.g., job burnout).

Tims et al. (2012) defined job crafting in the framework of the JD-R model as “changes that employees may make to balance their job demands and job resources with their personal abilities and needs” (p. 174). They also suggest four distinct job crafting dimensions: (1) increasing structural job resources (e.g., opportunity for development and autonomy), (2) increasing social job resources (e.g., feedback and social support), (3) increasing challenging job demands (e.g., responsibility and workload), and (4) decreasing hindering job demands (e.g., emotionally demanding interactions with others) (Tims et al., 2012). To encompass this broad range of job characteristics that employees may alter, in our study, we conceptualized job crafting within the JD-R model.

Job crafting is beneficial for both employees and organizations. Empirical studies have shown that job crafting behaviors are positively linked to employee well-being (conceptualized as high work engagement and job satisfaction, and low job burnout) (Tims et al., 2013), work meaning (Puchalska-Kamińska et al., 2019), person-job fit (Tims et al., 2016), and negatively linked to job boredom (Harju et al., 2016). Moreover, job crafting is related to higher work performance, and lower absenteeism (Ghitulescu, 2006) and turnover intentions (Rudolph et al., 2017). Knowing these positive consequences of job crafting, it is important to determine the antecedents of job crafting behaviors.

### Positive Emotions and Job Crafting

According to B&B theory, “experiences of positive emotions broaden people’s momentary thought-action repertoires, which in turn serves to build their enduring personal resources” (Fredrickson, 2001, p. 218). Fredrickson (2001) postulates that the effects of negative and positive emotions are complementary. Negative emotions narrow thought-action repertoires, promoting specific immediate behaviors, such as attack or escape. According to the *broaden hypothesis* positive emotions broaden the aforementioned repertoires, widening the scope of usual thoughts and actions, by including e.g., exploration or play (Fredrickson, 2001; Fredrickson and Branigan, 2005). Experimental studies have confirmed that positive emotions “broaden the scope of attention and thought-action repertoires” (Fredrickson and Branigan, 2005, p 313). Broaden hypothesis was also confirmed in the work context—research shown that positive emotions are related to proactive behaviors at work (Fritz and Sonnentag, 2007; Claes and van Loo, 2011). Building on broaden hypothesis (Fredrickson, 2001), we argue that because positive emotions at work broaden habitual modes of thinking and acting, they also predict job crafting behaviors.

Longitudinal studies conducted by Lu et al. (2014) and Tims et al. (2014b) confirmed a positive relationship between work engagement and job crafting over time. Authors of both studies refer to B&B theory (Fredrickson, 2001) and suggest that work engagement, as a positive motivational-affective state, may operate similarly to positive emotions and broaden employees’ thought-action repertoires. Moreover, the results of a meta-analysis of 122 independent samples confirmed that work engagement is strongly related to job crafting (Rudolph et al., 2017). However, to the best of our knowledge, no available longitudinal studies have examined the role of positive emotions at work as predictor of job crafting. Our study aims to fill this gap.

*Hypothesis 1*: Positive emotions at work predict job crafting dimensions, namely: (a) increasing structural job resources, (b) increasing social job resources, (c) increasing challenging job demands, and (d) decreasing hindering job demands.

### The Mediating Role of Self-Efficacy in the Relationship Between Positive Emotions and Job Crafting

The second part of the B&B Theory, the *build hypothesis*, posits that positive emotions build a variety of personal resources (Fredrickson, 2001). Numerous studies confirmed the relationship between positive emotions and personal resources, like hope, optimism, and self-efficacy (Fredickson et al., 2008; Ouweneel et al., 2011; Malinowski and Lim, 2015). Moreover, a diary study conducted by Xanthopoulou et al. (2012) demonstrated the link between daily positive emotions and daily personal resources (self-efficacy, self-esteem, and optimism). These results indicate that personal resources may be not only long-term but also immediate outcomes of positive emotions (Xanthopoulou et al., 2012). Bandura (2001), in his SCT, also suggests that experiencing positive emotions enhances one of the personal resources—self-efficacy. This resource is defined as “people’s beliefs about their capabilities to produce designated levels of performance that exercise influence over events that affect their lives” (Bandura, 1994, p. 72). Results of experimental studies confirmed the relationship between induced positive mood and self-efficacy (Kavanagh and Bower, 1985; Forgas et al., 1990). This relationship was also tested in the work context. A longitudinal study conducted by Laguna et al. (2017) demonstrated that positive job-related affect predicts work-related self-efficacy.

Tims et al. (2014a) suggest that self-efficacy predicts job crafting behaviors, because “self-efficacious employees may feel more confident that they are able to change aspects of their jobs. This confidence may, in turn, be related to actual job crafting behaviors (…)” (p. 493). This proposition is in line with SCT, according to which people with high self-efficacy beliefs are more involved in the process of selecting and changing their work environments (Wood and Bandura, 1989; Bandura, 1997). Results of a meta-analysis performed by Rudolph et al. (2017) confirmed that general self-efficacy predicts job crafting. Therefore, building on B&B theory (Fredrickson, 2001) and SCT (Bandura, 1997), we argue that self-efficacy acts as a mediator between positive emotions at work and job crafting behaviors. Self-efficacy is a domain-specific belief (Bandura, 1997, 2006). Because our study is conducted in a work context, we investigate the mediating role of *occupational self-efficacy*—“the competence that a person feels concerning the ability to successfully fulfill the tasks involved in his or her job” (Rigotti et al., 2008, p. 239).

*Hypothesis 2*: Occupational self-efficacy mediates the relationship between positive emotions at work and job crafting dimensions, namely: (a) increasing structural job resources, (b) increasing social job resources, (c) increasing challenging job demands, and (d) decreasing hindering job demands. High positive emotions at work would lead to higher occupational self-efficacy that, in turn, would predict higher job crafting.

## Study 1

### Methods

#### Participants and Procedure

This study was approved by the departmental ethics committee (decision number 17/2014). All subjects participated in the study voluntarily and, in accordance with the Declaration of Helsinki, granted informed consent. Employees who use information and communication technology (ICT) in their jobs on a daily basis were recruited to be participants. Recruitment was carried out online using social media platforms (e.g., Facebook, LindkedIn), recruitment ads on the university website and distributed through mailing lists of some companies that agreed to help in data collection. Questionnaires were administered online. Study participants had to meet the following criteria: (1) being at least 18 years old and (2) using ICT at daily work. The study had three-wave prospective design (with data being collected at three intervals, referred as T1, T2, and T3, respectively) with a time lag of approximately 2 months between each wave.

A convenience sample of 270 ICT workers completed T1 assessment and then was invited by e-mail to take part in T2 and T3 assessments. The questionnaires were filled out by 149 ICT workers at T2 (55.2% of the original sample) and 138 ICT workers at T3 (51.1% of the original sample). Participants with missing data of the dependent variable (job crafting dimensions) were excluded from the analysis. Results of Little’s test indicated that data were missing completely at random, χ^2^ = (23) = 23.48, *p* = 0.43. In the final sample (*N* = 124), 81.5% of the participants were female. The mean age was 33.93 years old (*SD* = 8.64). On average, participants were employed for 10.82 years (*SD* = 7.33), and worked 40.59 h per week (*SD* = 10.68). Most participants (73.4%) reported at least BA university degree.

#### Measures

*Positive emotions at work* were measured with the positive affect subscale of the Polish version of the Job-Related Affective Well-Being Scale (JAWS; Basińska et al., 2014), originally developed by Van Katwyk et al. (2000). JAWS consists of 20 items and two subscales to assess *positive affect* (10 items; e.g., “content,” “energetic”) and *negative affect* (10 items; e.g., “anxious,” “gloomy”). Participants indicated on a five-point scale from 1 (never) to 5 (very often) their frequency of experiencing certain positive emotions in their job. The internal consistency in the present study was: α = 0.91 (T1), α = 0.92 (T2), and α = 0.93 (T3).

*Job crafting* was measured using the Polish version of the Job Crafting Scale (JCS; Rogala and Cieślak, 2019), originally developed by Tims et al. (2012). JCS consists of 21 items and four subscales to assess *increasing structural job resources* (five items; α = 0.74 at T1, α = 0.76 at T2, and α = 0.80 at T3, e.g., “I try to develop myself professionally”), *increasing social job resources* (five items; α = 0.68 at T1, α = 0.76 at T2, and α = 0.77 at T3; e.g., “I ask colleagues for advice”), *increasing challenging job demands* (five items; α = 0.76 at T1, α = 0.74 at T2, and α = 0.84 at T3; e.g., “When there is not much to do at work, I see it as an opportunity to start new projects”), and *decreasing hindering job demands* (six items; α = 0.77 at T1, α = 0.71 at T2, and α = 0.76 at T3; e.g., “I make sure that my work is mentally less intense”). Participants had to indicate on a five-point scale ranging from 1 (never) to 5 (very often) how often they engage in each of the aforementioned behaviors.

*Occupational self-efficacy* was assessed with the Polish adaptation of a short version of the Occupational Self-Efficacy Scale (OSES; Rigotti et al., 2008), which consists of six items (e.g., “I can remain calm when facing difficulties in my job because I can rely on my abilities”). Participants were asked to evaluate items on a scale ranging from (1) totally disagree to (6) totally agree. The internal consistency in the present study was: α = 0.79 (T1), α = 0.82 (T2), and α = 0.82 (T3).

#### Data Analysis

To test our hypotheses, we performed regression analysis with bootstrapping using the PROCESS macro for IBM SPSS Statistics (Hayes, 2013). We applied model 4 (simple mediation) with 50,000 bootstrapped replications and estimated four independent mediation models for all four dimensions of job crafting. In our analyses, we used indirect effects based on bias-corrected 95% confidence intervals. Following the suggestions of MacKinnon et al. (2012) regarding longitudinal mediation models, the independent variable (positive emotions at work), the mediator (occupational self-efficacy), and the dependent variables (job crafting dimensions) were assessed at different time points (T1, T2, and T3). We also included baseline measures (T1) of the mediator and the dependent variable as covariates (MacKinnon et al., 2012).

### Results and Discussion

The correlations and descriptive statistics of study variables are presented in Table 1.

**TABLE 1:** Means, standard deviations, and correlations between variables in Study 1.

**Variable**	***M***	***SD***	**1**	**2**	**3**	**4**	**5**	**6**	**7**	**8**	**9**	**10**	**11**	**12**	**13**	**14**	**15**	**16**	**17**	**18**
(1) Positive emotions at work T1	2.92	0.76	–																	
(2) Increasing structural resources T1	4.20	0.55	0.31^∗∗^	–																
(3) Increasing social resources T1	2.62	0.75	0.20^∗^	0.07	–															
(4) Increasing challenging demands T1	3.27	0.72	0.18^∗^	0.50^∗∗^	0.20^∗^	–														
(5) Decreasing hindering demands T1	3.07	0.70	0.05	–0.17	–0.02	–0.11	–													
(6) Occupational self-efficacy T1	4.84	0.65	0.36^∗∗^	0.29^∗∗^	–0.01	0.40^∗∗^	–0.02	–												
(7) Positive emotions at work T2	2.84	0.79	0.71^∗∗^	0.37^∗∗^	0.13	0.26^∗∗^	0.04	0.27^∗∗^	–											
(8) Increasing structural resources T2	4.06	0.55	0.28^∗∗^	0.73^∗∗^	0.03	0.49^∗∗^	–0.15	0.32^∗∗^	0.39^∗∗^	–										
(9) Increasing social resources T2	2.58	0.77	0.18^∗^	0.06	0.72^∗∗^	0.14	–0.04	–0.04	0.16	0.07	−									
(10) Increasing challenging demands T2	3.23	0.73	0.20^∗^	0.56^∗∗^	0.13	0.62^∗∗^	−0.19^∗^	0.27^∗∗^	0.32^∗∗^	0.69^∗∗^	0.22^∗^	–								
(11) Decreasing hindering demands T2	3.10	0.64	0.01	–0.26^∗∗^	–0.01	–0.15	0.62^∗∗^	–0.01	–0.06	−0.21^∗^	0.08	–0.11	–							
(12) Occupational self-efficacy T2	4.72	0.67	0.24^∗∗^	0.30^∗∗^	–0.03	0.43^∗∗^	–0.08	0.62^∗∗^	0.30^∗∗^	0.39^∗∗^	–0.01	0.48^∗∗^	–0.03	–						
(13) Positive emotions at work T3	2.76	0.83	0.58^∗∗^	0.29^∗∗^	0.10	0.19^∗^	–0.04	0.27^∗∗^	0.60^∗∗^	0.32^∗∗^	0.16	0.27^∗∗^	–0.06	0.24^∗∗^	–					
(14) Increasing structural resources T3	3.99	0.66	0.32^∗∗^	0.59^∗∗^	0.13	0.51^∗∗^	–0.06	0.32^∗∗^	0.33^∗∗^	0.63^∗∗^	0.20^∗^	0.56^∗∗^	−0.19^∗^	0.32^∗∗^	0.48^∗∗^	–				
(15) Increasing social resources T3	2.51	0.80	0.17	0.03	0.56^∗∗^	0.16	0.02	0.03	0.15	0.09	0.71^∗∗^	0.12	0.09	0.04	0.30^∗∗^	0.27^∗∗^	–			
(16) Increasing challenging demands T3	3.16	0.82	0.31^∗∗^	0.53^∗∗^	0.18^∗^	0.65^∗∗^	–0.13	0.27^∗∗^	0.34^∗∗^	0.54^∗∗^	0.25^∗∗^	0.72^∗∗^	–0.16	0.37^∗∗^	0.49^∗∗^	0.75^∗∗^	0.35^∗∗^	–		
(17) Decreasing hindering demands T3	3.14	0.66	–0.09	–0.18	–0.06	−0.20^∗^	0.59^∗∗^	–0.11	–0.10	−0.20^∗^	0.06	–0.16	0.60^∗∗^	–0.10	–0.25^∗∗^	–0.24^∗∗^	–0.02	−0.21^∗^	–	
(18) Occupational self-efficacy T3	4.70	0.66	0.25^∗∗^	0.36^∗∗^	0.04	0.42^∗∗^	–0.05	0.60^∗∗^	0.32^∗∗^	0.33^∗∗^	0.00	0.39^∗∗^	–0.08	0.70^∗∗^	0.37^∗∗^	0.41^∗∗^	0.04	0.41^∗∗^	–0.12	–

**N* = 124; T1 = Time 1; T2 = Time 2; T3 = Time 3. ^∗^*p* < *0.05*. ^∗∗^*p* < 0.01.*

The results of the mediation analysis referring to both direct and indirect effects of positive emotions on job crafting (Hypotheses 1 and 2) are presented in Table 2. The direct effect of positive emotions at work (T1) on increasing structural resources (T3; Hypothesis 1a), increasing social resources (T3; Hypothesis 1b), and decreasing hindering demands (T3; Hypothesis 1d) was not significant. However, in line with Hypothesis 1c, the direct effect of positive emotions at work (T1) on increasing challenging demands (T3) was positive and significant; *B* = 0.23, SE = 0.08, 95% CI [0.07; 0.38]. A high level of positive emotions at work at T1 was associated with a high level of increasing challenging demands at T3. The indirect effects of positive emotions at work (T1) on all four job crafting dimensions—increasing structural resources (T3; Hypothesis 2a), increasing social resources (T3; Hypothesis 2b), increasing challenging demands (T3; Hypothesis 2c), and decreasing hindering demands (T3; Hypothesis 2d)—via occupational self-efficacy (T2; the mediator) were not significant. Hypotheses 2a–2d were all rejected.

**TABLE 2:** Mediation analysis in Study 1 (*N* = 124).

	***B***	***SE B***	**95% Cl LL**	**95% Cl UL**
**Positive emotions at work T1 → Occupational self-efficacy T2 → Increasing structural resources T3**				
Direct effect	0.09	0.07	–0.04	0.23
Indirect effect	–0.00	0.01	–0.03	0.01
**Positive emotions at work T1 → Occupational self-efficacy T2 → Increasing social resources T3**				
Direct effect	0.05	0.09	–0.12	0.23
Indirect effect	0.00	0.01	–0.01	0.03
**Positive emotions at work T1 → Occupational self-efficacy T2→Increasing challenging demands T3**				
Direct effect	**0.23**	0.08	0.07	0.38
Indirect effect	0.00	0.01	–0.02	0.03
**Positive emotions at work T1 → Occupational self-efficacy T2 → Decreasing hindering demands T3**				
Direct effect Indirect effect	–0.09	0.07	–0.22	0.05
	0.00	0.01	–0.01	0.02

*T1 = Time 1; T2 = Time 2; T3 = Time 3; LL = lower limit; CI = confidence interval; UL = upper limit. Unstandardized regression coefficients are reported. Values of coefficient presented in bold are significant. Bootstrap sample size = 50,000.*

The results of Study 1 suggest that positive emotions at work prospectively predict only one job crafting dimension—increasing challenging demands. Similar findings were reported by Petrou et al. (2012). Their findings revealed that increasing challenging demands was the only job crafting dimension positively associated with work engagement (Petrou et al., 2012). Results of Study 1 did not support the mediating role of occupational self–efficacy between positive emotions at work and job crafting dimensions. One possible explanation is that self-efficacy is a context-specific belief—which is highly probable to vary depending on the kind of activity it is connected with (Bandura, 1997, 2006). In our study, we investigated the role of occupational self-efficacy. Previous studies suggest that using more specific measures of self-efficacy allows to obtain more accurate results (e.g., Salanova et al., 2002). Thus, in future studies, even more specific self-efficacy measures should be used, i.e., job crafting self-efficacy (Roczniewska et al., unpublished).

Bandura (2006) argues that we should differentiate the source of the data (i.e., individual) and the level (individual or collective) of the measured phenomenon. In Study 1, we conceptualized all constructs at the individual level. Because contemporary organizations try to increase the flexibility and autonomy throughout their structures, employees often perform their duties not individually, but in work teams (see Colquitt et al., 2002; Salanova et al., 2003). Employees working in a team can share their emotions and beliefs (Salanova et al., 2014). Numerous studies confirmed the relationship between collective constructs, such as collective efficacy, with employees’ well-being and performance (Salanova et al., 2003; Stajkovic et al., 2009). To include teamwork context in Study 2, we conceptualize the independent variable and the mediator at the collective level. Both collective constructs are measured using referent-shift model by asking employees collectively formulated items (Chan, 1998), e.g., “I feel confident about the capability of my group to perform the tasks very well” (Salanova et al., 2003, p. 68). Moreover, to extend knowledge about the antecedents of job crafting, in Study 2, we verify the role of collective flow at work as a predictor of job crafting dimensions. Collective flow at work encompasses a wider context than positive emotions at work, including not only the area of activity to which emotions are related to (work), but also their source (high level of challenges and skills) (Llorens et al., 2013).

## Study 2

### Collective Flow and Job Crafting

Flow is an autotelic, optimal experience that results from high challenges balanced with adequate skills (Csikszentmilhalyi and Lefevre, 1989; Fong et al., 2014). It can be defined as a “peculiar dynamic state—the holistic sensation that people feel when they act with total involvement” (Csikszentmihalyi, 1975, p. 36). Salanova et al. (2014) suggest that “similar psychological process occurs at the group level” (p. 437). Collective flow can be defined as “a collective state that occurs when a group is performing at the peak of its abilities” (Sawyer, 2003, p. 167). In the work context, collective flow is described as a positive experience in a work setting characterized by collective absorption and collective enjoyment. Employees experiencing *collective flow at work* are highly concentered on the task, lose their sense of time, and have less awareness of self. Moreover, they share feelings of joy, elation, and enthusiasm (Salanova et al., 2014).

According to the broaden hypothesis of B&B theory, “positive emotions broaden habitual modes of thinking or acting” (Fredrickson, 2001, p. 220). Demerouti (2006) argues that because the construct of flow encompasses enjoyment, broaden hypothesis could also apply to flow at work. In her longitudinal study, she confirmed that flow at work predicts in-role and extra-role performance (Demerouti, 2006). Research has shown that flow at work is also associated with occupational success (Marzec, 2016). These results suggest that flow at work might operate similarly to positive emotions and broadens employees’ thought–action repertoires. Although, the aforementioned studies refer only to flow at work assessed at the individual, not collective level. Moreover, Parker et al. (2010) argue that flow at work can motivate proactive behaviors, such as job crafting. They suggest that “because challenge needs to be relatively high before flow is possible (…), individuals need increasingly greater challenge to experience flow. The desire for flow can therefore prompt proactive action, such as crafting a job to take on more difficult tasks” (Parker et al., 2010, p. 9). However, to the best of our knowledge, no previous longitudinal studies have examined the role of flow at work as a predictor of job crafting. Thus, building on B&B theory, we present the following hypothesis:

*Hypothesis 3*: Collective flow at work predicts job crafting dimensions, namely: (a) increasing structural job resources, (b) increasing social job resources, (c) increasing challenging job demands, and (d) decreasing hindering job demands.

### The Mediating Role of Collective Efficacy in the Relationship Between Collective Flow and Job Crafting

The build hypothesis of the B&B theory posits that “positive emotions set people on trajectories of growth that, over time, build consequential personal resources” (Fredickson et al., 2008, p. 2), such as self-efficacy. The role of positive emotions as predictors of self-efficacy beliefs is also in line with SCT (Bandura, 1997). Drawing upon both aforementioned theories, Salanova et al. (2006) suggest that being a positive experience, work-related flow builds employees self-efficacy. Results of their study confirmed the predictive role of flow at work in the context of self-efficacy beliefs. Similar relationships may be observed at the collective level. *Collective efficacy* is defined, in accordance with SCT, as a “group’s shared belief in its conjoint capabilities to organize and execute the courses of action required to produce given levels of attainments” (Bandura, 1997, p. 447). Salanova et al. (2014) demonstrated that collective flow at work predicts collective efficacy beliefs over time. What is more, Bandura (2001) argues that “people’s shared belief in their collective power to produce desired results is a key ingredient of collective agency” (p. 14). According to a meta-analysis conducted by Stajkovic et al. (2009), collective efficacy is positively related to group performance. In their meta-analysis, Rudolph et al. (2017) confirmed that general self-efficacy, assessed at the individual level, predicts job crafting. However, to the best of our knowledge, no available longitudinal studies have investigated the role of collective self-efficacy in the context of job crafting behaviors. Building on the B&B theory (Fredrickson, 2001) and SCT (Bandura, 1997), we predict the following:

*Hypothesis 4*: Collective efficacy beliefs mediate the relationship between collective flow at work and job crafting dimensions, namely: (a) increasing structural job resources, (b) increasing social job resources, (c) increasing challenging job demands, and (d) decreasing hindering job demands. High collective flow at work would be related to higher collective efficacy beliefs that, in turn, would lead to higher job crafting.

### Methods

#### Participants and Procedure

The departmental ethics committee approved the study (decision number 17/2014). Study participants were: (1) adults (18 and over years old), (2) who use ICT on a daily basis at work, and (3) work as a part of a team during the majority of the workweek. Informed consents were obtained from all subjects. Recruitment was carried out online, using nationwide research panel. In order to test our hypotheses, we conducted a three-wave prospective study. The time lag between each wave (T1, T2, and T3) was approximately 2 months.

The sample at T1 comprised of 248 participants who were also invited to fill out the questionnaires at T2 and T3. Out of all participants, 179 took part in the study at T2 (72.2% of the original sample) and 99 at T3 (39.9% of the original sample). All statistical analyses were performed with a sample of subjects who participated across three measurement points—cases with missing data were deleted listwise. Results of Little’s test showed that data were missing completely at random, χ^2^ = (13) = 11.99, *p* = 0.53. The final sample included in the analyses comprised 99 participants (51% female). The average age of the participants was 37.84 years (*SD* = 7.42). On average, the participants worked 40.93 h per week (*SD* = 14.96), including 35.29 h of work as a part of a team (*SD* = 12.87). Average tenure of participants was 15.94 years (*SD* = 8.42). Most participants had at least a BA university degree (66.7%).

#### Measures

*Collective flow at work* was measured using the Polish version of the Collective Flow Scale (CFS; Salanova et al., 2014). CFS consists of 10 items and two subscales to assess *collective absorption* (seven items; e.g., “Time flies when my group is working”) and *collective enjoyment* (three items; e.g., “The group members enjoy themselves while doing the task”). Participants had to indicate the frequency of their flow experiences with the group task using a seven-point scale ranging from 0 (never) to 6 (all the time). In our study, we used the total score to assess collective flow at work. The internal consistency in the present study was: α = 0.89 (T1), α = 0.94 (T2), and α = 0.95 (T3).

*Job crafting* was assessed with the Polish version of the JCS (Rogala and Cieślak, 2019) described in Study 1. The internal consistency was as follows: *increasing structural job resources* —α = 0.84 (T1), α = 0.89 (T2), and α = 0.88 (T3); *increasing social job resources* —α = 0.86 (T1), α = 0.89 (T2), and α = 0.86 (T3); *increasing challenging job demands*—α = 0.81 (T1), α = 0.89 (T2), and α = 0.87 (T3); *decreasing hindering job demands*—α = 0.79 (T1), α = 0.85 (T2), α = 0.77 (T3).

*Collective efficacy beliefs* were measured using the Polish adaptation of the Perceived Collective Efficacy Scale (PCES; Salanova et al., 2003), which consists of four items (e.g., “My group is able to solve difficult task if we invest the necessary effort”). PCES has a six-point scale ranging from 0 (never) to 6 (always). The internal consistency in the present study was: α = 0.94 (T1), α = 0.96 (T2), and α = 0.97 (T3).

#### Data Analysis

To test our hypotheses in Study 2, we performed analyses equivalent to those described in Study 1—regression analysis with bootstrapping using the PROCESS macro (Hayes, 2013; model 4 with 50,000 bootstrapped replications). The independent variable (collective flow at work), the mediator (collective efficacy beliefs), and the dependent variable (job crafting dimensions) were subject to measurements at different time points, respectively—T1, T2, and T3. Baseline measures (T1) of the mediator and the dependent variable were also included as covariates (MacKinnon et al., 2012).

### Results and Discussion

Table 3 shows the descriptive statistics and correlations among study variables.

**TABLE 3:** Means, standard deviations, and correlations between variables in Study 2.

**Variable**	***M***	***SD***	**1**	**2**	**3**	**4**	**5**	**6**	**7**	**8**	**9**	**10**	**11**	**12**	**13**	**14**	**15**	**16**	**17**	**18**
(1) Collective flow at work T1	3.61	0.84	–																	
(2) Increasing structural resources T1	3.91	0.64	0.52^∗∗^	–																
(3) Increasing social resources T1	2.97	0.88	0.60^∗∗^	0.30^∗∗^	–															
(4) Increasing challenging demands T1	3.29	0.71	0.67^∗^	0.57^∗∗^	0.65^∗∗^	–														
(5) Decreasing hindering demands T1	3.42	0.62	0.41^∗∗^	0.38^∗∗^	0.52^∗∗^	0.43^∗∗^	–													
(6) Collective efficacy beliefs T1	4.52	0.91	0.51^∗∗^	0.52^∗∗^	0.23^∗^	0.32^∗∗^	0.15	–												
(7) Collective flow at work T2	3.35	1.02	0.56^∗∗^	0.41^∗∗^	0.27^∗∗^	0.46^∗∗^	0.34^∗∗^	0.39^∗∗^	–											
(8) Increasing structural resources T2	3.78	0.78	0.29^∗∗^	0.58^∗∗^	0.01	0.26^∗^	0.15	0.38^∗∗^	0.60^∗∗^	–										
(9) Increasing social resources T2	2.84	0.91	0.45^∗∗^	0.34^∗∗^	0.53^∗∗^	0.50^∗∗^	0.29^∗∗^	0.33^∗∗^	0.53^∗∗^	0.39^∗∗^	–									
(10) Increasing challenging demands T2	3.20	0.87	0.43^∗∗^	0.54^∗∗^	0.30^∗∗^	0.61^∗∗^	0.26^∗∗^	0.27^∗∗^	0.65^∗∗^	0.67^∗∗^	0.72^∗∗^	–								
(11) Decreasing hindering demands T2	3.41	0.70	0.18	0.29^∗∗^	0.12	0.06	0.35^∗∗^	0.21^∗^	0.50^∗∗^	0.63^∗∗^	0.53^∗∗^	0.53^∗∗^	–							
(12) Collective efficacy beliefs T2	4.27	1.10	0.35^∗∗^	0.45^∗∗^	0.04	0.18	0.06	0.58^∗∗^	0.58^∗∗^	0.76^∗∗^	0.37^∗∗^	0.48^∗∗^	0.59^∗∗^	–						
(13) Collective flow at work T3	3.44	1.06	0.60^∗∗^	0.59^∗∗^	0.36^∗∗^	0.53^∗∗^	0.28^∗∗^	0.48^∗∗^	0.61^∗∗^	0.45^∗∗^	0.54^∗∗^	0.58^∗∗^	0.31^∗∗^	0.44^∗∗^	–					
(14) Increasing structural resources T3	3.75	0.73	0.33^∗∗^	0.73^∗∗^	0.04	0.31^∗∗^	0.22^∗^	0.42^∗∗^	0.35^∗∗^	0.55^∗∗^	0.25^∗^	0.45^∗∗^	0.25^∗^	0.38^∗∗^	0.57^∗∗^	–				
(15) Increasing social resources T3	2.92	0.84	0.53^∗∗^	0.37^∗∗^	0.60^∗∗^	0.50^∗∗^	0.38^∗∗^	0.28^∗∗^	0.38^∗∗^	0.18	0.64^∗∗^	0.44^∗∗^	0.30^∗∗^	0.11	0.55^∗∗^	0.31^∗∗^	–			
(16) Increasing challenging demands T3	3.20	0.81	0.64^∗∗^	0.59^∗∗^	0.43^∗∗^	0.74^∗∗^	0.33^∗∗^	0.37^∗∗^	0.47^∗∗^	0.37^∗∗^	0.52^∗∗^	0.65^∗∗^	0.18	0.26^∗∗^	0.66^∗∗^	0.57^∗∗^	0.67^∗∗^	–		
(17) Decreasing hindering demands T3	3.38	0.59	0.21^∗^	0.47^∗∗^	0.15	0.14	0.47^∗∗^	0.19	0.24^∗^	0.32^∗∗^	0.17	0.22^∗^	0.42^∗∗^	0.17	0.33^∗∗^	0.56^∗∗^	0.39^∗∗^	0.39^∗∗^	–	
(18) Collective efficacy beliefs T3	4.20	1.21	0.40^∗∗^	0.53^∗∗^	0.13	0.21^∗^	0.11	0.62^∗∗^	0.37^∗∗^	0.46^∗∗^	0.29^∗∗^	0.33^∗∗^	0.24^∗^	0.56^∗∗^	0.63^∗∗^	0.70^∗∗^	0.24^∗^	0.37^∗∗^	0.35^∗∗^	–

**N* = 99; T1 = Time 1; T2 = Time 2; T3 = Time 3. ^∗^*p* < *0.05*. ^∗∗^*p* < 0.01.*

As can be observed in Table 4, the direct effect of collective flow at work (T1) on increasing structural resources (T3; Hypothesis 3a) and decreasing hindering demands (T3; Hypothesis 3d) was not significant. However, in line with Hypothesis 3b, the direct effect of collective flow at work (T1) on increasing social resources (T3) was significant; *B* = 0.24, SE = 0.11, 95% CI [0.01; 0.47]. What is more, in line with Hypothesis 3c, the direct effect of collective flow at work (T1) on increasing challenging demands was also significant; *B* = 0.21, SE = 0.09, 95% CI [0.02; 0.40]. High level of collective flow at work at T1 was related to high levels of both increasing social resources at T3 and increasing challenging demands at T3.

**TABLE 4:** Mediation analysis in Study 2 (*N* = 99).

	***B***	***SE B***	**95% Cl LL**	**95% Cl UL**
**Collective flow at work T1 → Collective efficacy beliefs T2 → Increasing structural resources T3**				
Direct effect	–0.09	0.07	–0.24	0.06
Indirect effect	0.00	0.01	–0.02	0.03
**Collective flow at work T1 → Collective efficacy beliefs T2 → Increasing social resources T3**				
Direct effect	**0.24**	0.11	0.01	0.47
Indirect effect	–0.01	0.03	–0.08	0.03
**Collective flow at work T1 → Collective efficacy beliefs T2 → Increasing challenging demands T3**				
Direct effect	**0.21**	0.09	0.02	0.40
Indirect effect	0.01	0.02	–0.01	0.07
**Collective flow at work T1 → Collective efficacy beliefs T2 → Decreasing hindering demands T3**				
Direct effect	–0.04	0.08	–0.20	0.11
Indirect effect	0.01	0.02	–0.01	0.06

*T1 = Time 1; T2 = Time 2; T3 = Time 3; LL = lower limit; CI = confidence interval; UL = upper limit. Unstandardized regression coefficients are reported. Values of coefficient presented in bold are significant. Bootstrap sample size = 50,000.*

The results also showed that indirect effect of collective flow at work (T1) on all four job crafting dimensions—increasing structural resources (T3), increasing social resources (T3), increasing challenging demands (T3), and decreasing hindering demands (T3)—via collective efficacy at work (T2; the mediator), was not significant. Thus, Hypotheses 4a–4d were rejected.

The results of Study 2 indicate that collective flow at work predicts two of job crafting dimensions—increasing social resources and increasing challenging demands. These results expand job crafting theory by demonstrating that different forms of job crafting may have unique antecedents. The dimension of increasing social resources refers to resources such as “social support, supervisory coaching, and feedback” (Tims et al., 2012, p. 176). Work enjoyment, the affective component of flow at work, was found to be positively associated with social support by colleagues (Bakker, 2005, 2008), performance feedback (Bakker, 2005), and social support orientation, or “the extent to which there are kindly and supportive relationships among organizational members”(Salanova et al., 2006, p. 8). Although individual and collective flow are similar, the latter construct has some unique characteristics. Employees working in teams influence each other—both positive and negative emotions can be transferred from one employee to the other in a non-conscious process of emotional contagion (Bakker et al., 2006). Emotional contagion is “the tendency to automatically mimic and synchronize facial expressions, vocalizations, postures, and movements with those of another person and, consequently, to converge emotionally” (Hatfield et al., 1993, p. 5). Several studies have confirmed emotional contagion in work teams (Bakker et al., 2006; Bakker and Xanthopoulou, 2009). Salanova et al. (2014) suggest that flow can become a collective social experience as a result of emotional contagion process during which employees synchronize with each other physically and emotionally (Hatfield et al., 1993). It is, therefore, possible that this synchronization encourages employees to increase their social resources by asking for support, coaching, or feedback. Regarding the dimension of increasing challenging demands, Parker et al. (2010) suggest that because a relatively high level of challenges is required for the flow to occur, employees engage in proactive behaviors and seek challenges to experience flow at work in the future. The results of Study 2 are in line with the aforementioned proposition. What is more, the results of several studies also confirmed that flow at work is positively associated with challenging job demands (Rodríguez-Sánchez et al., 2011; Llorens et al., 2013).

Results of Study 2 did not support the role of collective efficacy at work as a mediator of the relationship between collective flow at work and job crafting dimensions. One of the possible explanations might be that we applied too general measure of self-efficacy, that refer to working as a part of a team (Salanova et al., 2014). Because using context-specific self-efficacy measures allows to predict the outcomes more successfully (e.g., Salanova et al., 2002), future studies need to use measure that refer to job crafting behaviors (Roczniewska et al., unpublished). What is more, according to the extended channel model of flow, not only the challenges and skills combination predicts flow, but also self-efficacy beliefs. A study conducted by Rodríguez-Sánchez et al. (2011) confirmed the predictive role of self-efficacy in the context of flow at work. It is, therefore, possible that self-efficacy might act as a predictor of flow experience, not as a mediator in the collective flow at work–job crafting relationship. In the aforementioned study (Rodríguez-Sánchez et al., 2011), both self-efficacy and flow were measured at the individual level. Although the results of a study conducted by Salanova et al. (2014) confirmed that “collective efficacy beliefs predict collective flow over time, both being related reciprocally” (p. 435), the model tested in our study was based on assumptions derived from particular theories—B&B theory and SCT—which suggest unidirectional predictive role of collective flow (Bandura, 1997; Fredrickson, 2001). Moreover, the results of analyses conducted *post hoc* using data from Study 2 did not support the role of collective efficacy as a predictor of collective flow at work or any of the job crafting dimensions.

## General Discussion

### Study Contribution

Job crafting is a self-initiated behavior that has many positive effects on the functioning of employees and organizations (Rudolph et al., 2017). Representatives of many professions craft their job, e.g., hospital janitors (Wrzesniewski and Dutton, 2001), miners and manufacturers (De Beer et al., 2016), teachers (Ghitulescu, 2006), or police officers (Petrou et al., 2012; Roczniewska and Bakker, 2016). However, the evidence for the predictors of job crafting is limited. Results of previous studies indicate that several organizational factors predict job alterations, e.g., task complexity (Ghitulescu, 2006), task independence (Wrzesniewski and Dutton, 2001), job autonomy (Roczniewska and Puchalska-Kamińska, 2017), or perceived opportunity to craft (van Wingerden and Niks, 2017). Also, personal characteristics, such as proactive personality and promotion regulatory focus, were found to be related to overall job crafting (Rudolph et al., 2017). Research has also shown that specific job crafting dimensions can have different predictors. For example, promotion focus is related to increasing job resources (structural and social) and challenging job demands, whereas prevention focus is linked with decreasing hindering job demands (Lichtenthaler and Fischbach, 2018). Openness to experience is positively associated with increasing structural resources and increasing challenging demands. On the other hand, neuroticism and decreasing hindering job demands are positively related to each other (Rudolph et al., 2017). The studies described in the present article also suggest that different job crafting dimensions may have unique antecedents.

The first contribution of the studies is that they provide support for the broaden hypothesis of the B&B theory (Fredrickson, 2001) in the context of job crafting behaviors. Results of Study 1 suggest that positive emotions at work predict increasing challenging demands (e.g., initiating new projects) in the future, whereas results of Study 2 indicate the predictive role of collective flow at work in the context of increasing challenging demands and increasing social resources (e.g., asking for feedback). These findings highlight the role of positive work-related emotions as antecedents of different job crafting dimensions. What is more, the results of both aforementioned studies indicate that positive emotions may encourage employees to look for new challenges at work, but do not inspire them to increase their structural resources and reduce hindering job demands. Fredrickson and Branigan (2005) argue that positive emotions widen the scope of usual thoughts and actions by adding to the typical repertoire, e.g., play or explore. One possible explanation of these results is the similarity between the dimension of increasing challenging demands and exploratory behaviors—searching for new challenges employees get involved in new, previously unknown activities.

Results of Study 2 suggest that collective flow at work predicts increasing social resources in the follow-up. A similar relationship was not found for positive emotions at work and increasing social resources in Study 1. A potential explanation is that flow at work in Study 2 was assessed as a collective social experience, that may be a result of emotional contagion process (Salanova et al., 2014). It is, therefore, possible that employees working as a part of a team, who share their positive emotions with colleagues, may be more eager to increase their social resources, e.g., by asking for support. Although in line with the JD-R model decreasing hindering job demands is one of the job crafting dimensions (Tims et al., 2012), behaviors that include reducing demands (e.g., minimizing contact with certain people, avoiding making difficult decisions) are rather related to narrowing, not broadening thought–action repertoires. It is possible that this is why positive emotions at work (Study 1) and collective flow at work (Study 2) may not act as predictors of this particular job crafting dimension. Tims et al. (2012) proposed that “underlying processes that motivate employees to increase their job resources and challenging demands may be different from the processes that motivate employees to decrease their hindering job demands” (p. 183). What is more, Lichtenthaler and Fischbach (2018) suggested that we can distinguish between two types of job crafting behaviors. One is called promotion-focused job crafting (increasing resources and challenging demands), and the other prevention-focused job crafting (decreasing hindering demands). Results of two recent meta-analyses (Rudolph et al., 2017; Lichtenthaler and Fischbach, 2018) confirmed that only promotion-focused job crafting was positively related to work engagement and negatively associated with job burnout. One the other hand, prevention-focused job crafting and job strain were positively related to each other. Therefore, it might be that promotion- and prevention-focused job crafting have different predictors and outcomes, and the broaden hypothesis of the B&B theory (Fredrickson, 2001) should be tested only in the context of the first one.

Moreover, positive emotions are rarely related to a high level of hindering demands. Research has confirmed that positive emotions at work are negatively linked to job demands (Miles et al., 2002). According to the conservation of resources theory, the role of resources is to help in coping with demands and avoiding negative consequences (Hobfoll, 1989). What is more, according to the buffering hypothesis of the JD-R model, job resources buffer the negative consequences of job demands on employees’ well-being (Bakker and Demerouti, 2007). It might be that a high level of positive emotions at work is possible only when the level of hindering job demands is low. Employees, when their level of hindering demands is low, may be more eager to search for new challenges than to increase resources that may help them deal with the aforementioned demands. It is one possible explanation why positive work-related emotions assessed in both studies did not predict the dimension of increasing structural resources. Another possible explanation of these results may be that job crafting is a process that has specific dynamics. Harju et al. (2016) suggest that “after taking on new challenges in their jobs, employees might need to increase their job resources to deal with the increased (challenging) job demands” (p. 13). Results of their longitudinal study confirmed that increasing challenging demands predicts increasing both types of resources (Harju et al., 2016). Therefore, seeking challenges may initiate a gain cycle leading to other job crafting behaviors in the future.

Previous research has confirmed the broaden hypothesis in the context of breadth of attentional selection (Rowe et al., 2007) or “scope of attention and thought-action repertoires” (Fredrickson and Branigan, 2005, p. 313). However, the B&B theory describes the general mechanism of positive emotions, not related to any domain of human functioning. Empirical evidence for this hypothesis in the context of proactive behaviors is scarce (Fritz and Sonnentag, 2007; Claes and van Loo, 2011). No previous longitudinal studies have also investigated the role of positive emotions at work and collective flow at work as predictors of job crafting dimensions. Studies described in this paper provide additional evidence for the broaden hypothesis and extend knowledge on the predictive role of positive emotions in the work context. What is more, they extend the B&B theory because they further specify the kinds of behaviors that may be the outcomes of positive work-related emotions—increasing challenging demands (Studies 1 and 2) and increasing social resources (Study 2).

According to SCT, self-efficacy beliefs have four sources: mastery experiences, vicarious experiences, social persuasions, and emotional and physiological states (Bandura, 1997). In our studies, integrating the build hypothesis of the B&B theory (Fredrickson, 2001) and SCT (Bandura, 2001), we suggested that self-efficacy mediates the relationship between positive emotions at work and job crafting behaviors. Results showed that neither occupational self-efficacy (Study 1) nor collective efficacy beliefs (Study 2) mediates the aforementioned relationship. Therefore, it is possible that both measures of positive emotions do not act as predictors of self-efficacy beliefs. These results are in line with previous studies which suggest that mastery experiences are the most powerful source of self-efficacy across domains, whereas the correlation between emotional and physiological states and self-efficacy is rather weak (Usher and Pajares, 2008). Hence, our studies enhance the understanding of positive emotions at work as sources of self-efficacy beliefs.

### Limitations and Suggestions for Future Research

Several limitations to our studies need to be acknowledged. First, in both studies, we used self-reported measures, which may lead to common method variance (Spector, 2006). However, we have taken steps to minimize this method bias by collecting data at three points in time and using different scale ranges (Podsakoff et al., 2003). Future studies could also minimize this bias and use different types of measures, e.g., peer-ratings and supervisor assessments (Tims et al., 2012). A second limitation is related to the generalizability of our findings. We collected data among a relatively small and convenience sample of Polish employees who use ICT in their jobs on a daily basis. Moreover, in both studies, most participants reported at least a bachelor degree. This may limit the generalizability of the results. We encourage further research to replicate our findings with different samples.

According to the build hypothesis of the B&B theory (Fredrickson, 2001), positive emotions “build (…) enduring personal resources” (p. 218) over time. Although the results of previous studies suggest that positive emotions can build resources even on a daily basis (Xanthopoulou et al., 2012), 2 months interval between measurement times in our studies could be too short to observe this building effect. Future studies may want to further investigate building hypothesis in the context of self-efficacy beliefs, choosing different time lags. Because of the context-specific nature of self-efficacy, future studies on job crafting should also use more specific measures to assess job-crafting self-efficacy, defined as “individual’s beliefs about his or her own capability to modify demands and resources present at their job to better fit their needs and preferences” (Roczniewska et al., unpublished, p. 2). Results of a study conducted by Roczniewska et al., unpublished confirmed that job-crafting self-efficacy is a more accurate predictor of job-crafting behaviors than general self-efficacy.

Our studies focused on the direct and indirect effects of positive work-related emotions. However, empirical studies have confirmed reciprocal relationships between positive emotions at work and self-efficacy beliefs (Salanova et al., 2011; Laguna et al., 2017) and between collective flow at work and collective efficacy beliefs (Salanova et al., 2014). Therefore, future studies could investigate dynamic and reciprocal relationships between work-related emotions, self-efficacy beliefs, and job crafting dimensions over time. In both our studies, we expected that positive emotions at work will have an effect on all job crafting dimensions. Harju et al. (2016) suggest that job crafting is a process in which increasing challenging demands predict increasing both types of resources. Thus, it is possible that, by predicting increasing challenging demands, positive emotions at work might trigger a gain cycle. These findings require further research attention. Moreover, research suggest that decreasing hindering job demands may operate differently than other job crafting dimensions (Rudolph et al., 2017; Lichtenthaler and Fischbach, 2018). Future studies need to explore predictors of this specific job crafting behavior. Further studies regarding the role of negative emotions in the context of job crafting would also be worthwhile.

## Conclusion and Practical Implications

The studies described in this paper highlight the role of positive work-related emotions as predictors of different job crafting dimensions measured in the follow-up. Employees that experience positive emotions at work and collective flow at work are more eager to increase challenging demands as a job crafting behavior. Research confirmed that dealing with challenges has a positive effect on performance (LePine et al., 2005). What is more, the results of our studies suggest that collective flow at work also predicts increasing social resources as a job crafting behavior. Therefore, employees who experience flow at the collective level are more eager to ask for help or feedback from their supervisors and colleagues. Results of previous research indicate that dimensions of increasing challenging demands and increasing social resources are positively associated with job satisfaction, work engagement, and performance (Rudolph et al., 2017). Thus, organizations may be interested in creating an organizational climate that promotes experiencing positive emotions. Results of our research also point to propitious directions for interventions to boost job crafting among employees. These could include techniques that enhance positive affect, e.g., loving-kindness meditation (Fredickson et al., 2008). According to affective events theory, the occurrence of positive events during the work also stimulates positive emotions (Weiss and Cropanzano, 1996).

## Data Availability Statement

The datasets generated for this study are available on request to the corresponding author.

## Ethics Statement

The studies involving human participants were reviewed and approved by Ethics Committee of the Faculty of Psychology, SWPS University of Social Sciences and Humanities, Warsaw, Poland. The patients/participants provided their written informed consent to participate in this study.

## Author Contributions

Both authors contributed to conception and design of the study and wrote the first draft of the manuscript. AR organized the database and performed the statistical analysis.

## Conflict of Interest

The authors declare that the research was conducted in the absence of any commercial or financial relationships that could be construed as a potential conflict of interest.

## References

[B1] Bakker A. B. (2005). Flow among music teachers and their students: the crossover of peak experiences. *J. Vocat. Behav.* 66 26–44. 10.1016/j.jvb.2003.11.001

[B2] Bakker A. B. (2008). The work-related flow inventory: construction and initial validation of the WOLF. *J. Vocat. Behav.* 72 400–414. 10.1016/j.jvb.2007.11.007

[B3] Bakker A. B. Demerouti E. (2007). The job demands−resources model: state of the art. *J. Manag. Psychol.* 22 309–328. 10.1108/02683940710733115

[B4] Bakker A. B. Emmerik H. V. Euwema M. C. (2006). Crossover of burnout and engagement in work teams. *Work Occup.* 33 464–489. 10.1177/0730888406291310

[B5] Bakker A. B. Xanthopoulou D. (2009). The crossover of daily work engagement: test of an actor-partner interdependence model. *J. Appl. Psychol.* 94 1562–1571. 10.1037/a0017525 19916663

[B6] Bandura A. (1994). “Self-efficacy,” in *Encyclopedia of Human Behavior*, ed. Ramachaudran V. S. (New York, NY: Academic Press), 71–81.

[B7] Bandura A. (1997). *Self-Efficacy: the Exercise of Control.* New York, NY: W.H. Freeman.

[B8] Bandura A. (2001). Social cognitive theory: an agentic perspective. *Annu. Rev. Psychol.* 52 1–26. 10.1146/annurev.psych.52.1.1 11148297

[B9] Bandura A. (2006). “Guide for constructing self-efficacy scales,” in *Self-Efficacy Beliefs of Adolescents*, eds Pajares F. Urdan T. (Greenwich: CT: Information Age Publishing), 307–337.

[B10] Basińska B. A. Gruszczyńska E. Schaufeli W. B. (2014). Psychometric properties of the polish version of the job-related affective well-being scale. *Int. J. Occup. Med. Environ. Health* 27 993–1004. 10.2478/s13382-014-0329-x 25503895

[B11] Chan D. (1998). Functional relations among constructs in the same content domain at different levels of analysis: a typology of composition models. *J. Appl. Psychol.* 83 234–246. 10.1037/0021-9010.83.2.234

[B12] Claes R. van Loo K. (2011). Relationships of proactive behaviour with job-related affective well-being and anticipated retirement age: an exploration among older employees in Belgium. *Eur. J. Ageing* 8 233–241. 10.1007/s10433-011-0203-7 28798653 PMC5547361

[B13] Colquitt J. A. Hollenbeck J. R. Ilgen D. R. LePine J. A. Sheppard L. (2002). Computer-assisted communication and team decision-making performance: the moderating effect of openness to experience. *J. Appl. Psychol.* 87 402–410. 10.1037/0021-9010.87.2.402 12002966

[B14] Csikszentmihalyi M. (1975). *Beyond Boredom and Anxiety.* San Francisco, CA: Jossey-Bass Publishers.

[B15] Csikszentmilhalyi M. Lefevre J. (1989). Optimal experience in work and leisure. *J. Pers. Soc. Psychol.* 56 815–822. 10.1037//0022-3514.56.5.815 2724069

[B16] De Beer L. T. Tims M. Bakker A. B. (2016). Job crafting and its impact on work engagement and job satisfaction in mining and manufacturing. *South African J. Econ. Manag. Sci.* 19 400–412. 10.17159/2222-3436/2016/v19n3a7

[B17] Demerouti E. (2006). Job characteristics, flow, and performance: the moderating role of conscientiousness. *J. Occup. Health Psychol.* 11 266–280. 10.1037/1076-8998.11.3.266 16834474

[B18] Fong C. J. Zaleski D. J. Leach J. K. (2014). The challenge–skill balance and antecedents of flow: a meta-analytic investigation. *J. Posit. Psychol.* 10 425–446. 10.1080/17439760.2014.967799

[B19] Forgas J. P. Bower G. H. Moylan S. J. (1990). Praise or blame? Affective influences on attributions for achievement. *J. Pers. Soc. Psychol.* 59 809–819. 10.1037/0022-3514.59.4.809 2254856

[B20] Fredickson B. L. Cohn M. A. Coffey K. A. Pek J. Finkel S. M. (2008). Open hearts build lives: positive emotions, induced through loving-kindness meditation, build consequential personal resources. *J. Pers. Soc. Psychol.* 95 1045–1062. 10.1037/a0013262.Open 18954193 PMC3156028

[B21] Fredrickson B. L. (2001). The role of positive emotions in positive psychology. The Broaden-and-Built theory of positive emotions. *Am. Psychol.* 56 218–226. 10.1O37//0OO3-O66X.56.3.218 11315248 PMC3122271

[B22] Fredrickson B. L. Branigan C. (2005). Positive emotions broaden the scope of attention and thought-action repertoires. *Cogn. Emot.* 19 313–332. 10.1080/02699930441000238 21852891 PMC3156609

[B23] Fritz C. Sonnentag S. (2007). Antecedents of day-level proactive behavior: a look at job stressors and positive affect during the workday. *J. Manage.* 35 94–111. 10.1177/0149206307308911

[B24] Ghitulescu B. (2006). *Shaping Tasks and Relationships at Work: Examining the Antecedents and Consequences of Employee Job Crafting.* Doctoral Dissertation, University of Pittsburgh, Pittsburgh.

[B25] Harju L. K. Hakanen J. J. Schaufeli W. B. (2016). Can job crafting reduce job boredom and increase work engagement? A three-year cross-lagged panel study. *J. Vocat. Behav.* 9 11–20. 10.1016/j.jvb.2016.07.001

[B26] Hatfield E. Cacioppo J. T. J. Rapson R. L. (1993). Emotional contagion. *Curr. Dir. Psychol. Sci.* 2 96–99. 10.1111/1467-8721.ep10770953

[B27] Hayes A. (2013). *Introduction to Mediation, Moderation, and Conditional Process Analysis.* New York, NY: Guilford Press.

[B28] Hobfoll S. E. (1989). Conservation of resources: a new attempt at conceptualizing stress. *Am. Psychol.* 44 513–524. 10.1037//0003-066X.44.3.513 2648906

[B29] Kavanagh D. J. Bower G. H. (1985). Mood and self-efficacy: Impact of joy and sadness on perceived capabilities. *Cognit. Ther. Res.* 9 507–525. 10.1007/BF01173005

[B30] Laguna M. Razmus W. Żaliński A. (2017). Dynamic relationships between personal resources and work engagement in entrepreneurs. *J. Occup. Organ. Psychol.* 90 248–269. 10.1111/joop.12170

[B31] LePine J. A. Podsakoff N. P. LePine M. A. (2005). A meta-analytic test of the challenge Stressor-hindrance stressor framework: an explanation for inconsistent relationships among Stressors and performance. *Acad. Manag. J.* 48 764–775. 10.5465/AMJ.2005.18803921

[B32] Lichtenthaler P. W. Fischbach A. (2018). A meta-analysis on promotion- and prevention- focused job crafting. *Eur. J. Work Organ. Psychol.* 00 1–21. 10.1080/1359432X.2018.1527767

[B33] Llorens S. Salanova M. Rodríguez A. M. (2013). How is flow experienced and by whom? Testing flow among occupations. *Stress Heal.* 29 125–137. 10.1002/smi.2436 22674654

[B34] Lu C. Q. Wang H. Lu J. J. Du D. Bakker A. B. (2014). Does work engagement increase person-job fit? The role of job crafting and job insecurity. *J. Vocat. Behav.* 84 142–152. 10.1016/j.jvb.2013.12.004

[B35] Lyons P. (2008). The crafting of jobs and individual differences. *J. Bus. Psychol.* 23 25–36. 10.1007/s10869-008-9080-2

[B36] MacKinnon D. P. Cheong J. Pirlott A. G. (2012). ““Statistical Medition Analysis,” in APA handbook of research methods,” in *Research designs: Quantitative, Qualitative, Neuropsychological, and Biological*, Vol. 2 eds Cooper K. J. Camic H. Long P. M. Panter D. L. Rindskopf A. T. Sher D. (Washington, DC: American Psychological Association).

[B37] Malinowski P. Lim H. J. (2015). Mindfulness at work: positive affect, hope, and optimism mediate the relationship between dispositional mindfulness, work engagement, and well-being. *Mindfulness.* 6 1250–1262. 10.1007/s12671-015-0388-5

[B38] Marzec I. (2016). Poczucie związanego z pracą przepływu jako czynnik sukcesu zawodowego pracowników w sektorze publicznym. *Stud. Ekon.* 270 200–208.

[B39] Miles D. E. Borman W. E. Spector P. E. Fox S. (2002). Building an integrative model of extra role work behaviors: a comparison of counterproductive work behavior with organizational citizenship behavior. *Int. J. Sel. Assess.* 10 51–57. 10.1111/1468-2389.00193

[B40] Ouweneel E. Le Blanc P. M. Schaufeli W. B. (2011). Flourishing students: a longitudinal study on positive emotions, personal resources, and study engagement. *J. Posit. Psychol.* 6 142–153. 10.1080/17439760.2011.558847

[B41] Parker S. K. Bindl U. K. Strauss K. (2010). Making Things Happen: a Model Of Proactive Motivation. *J. Manage.* 36 827–856. 10.1177/0149206310363732

[B42] Petrou P. Demerouti E. Peeters M. C. W. Schaufeli W. B. Hetland J. (2012). Crafting a job on a daily basis: contextual correlates and the link to work engagement. *J. Organ. Behav.* 33 1120–1141. 10.1002/job.1783

[B43] Petrou P. Demerouti E. Schaufeli W. B. (2015). Job crafting in changing organizations: antecedents and implications for exhaustion and performance. *J. Occup. Health Psychol.* 20 470–480. 10.1037/a0039003 25798717

[B44] Podsakoff P. M. MacKenzie S. B. Lee J. Y. Podsakoff N. P. (2003). Common method biases in behavioral research: a critical review of the literature and recommended remedies. *J. Appl. Psychol.* 88 879–903. 10.1037/0021-9010.88.5.879 14516251

[B45] Puchalska-Kamińska M. Czerw A. Roczniewska M. (2019). Work meaning in self and world perspective: new outlook on the WAMI Scale. *Soc. Psychol. Bull.* 14 1–29. 10.32872/spb.v14i1.30207

[B46] Rigotti T. Schyns B. Mohr G. (2008). A short version of the occupational self-efficacy scale: structural and construct validity across five countries. *J. Career Assess.* 16 238–255. 10.1177/1069072707305763

[B47] Roczniewska M. Bakker A. B. (2016). Who seeks job resources, and who avoids job demands? The link between dark personality traits and job crafting. *J. Psychol.* 150 1026–1045. 10.1080/00223980.2016.1235537 27719551

[B48] Roczniewska M. A. Puchalska-Kamińska M. (2017). Are managers also ‘crafting leaders’? The link between organizational rank, autonomy, and job crafting. *Polish Psychol. Bull.* 48 198–211. 10.1515/ppb-2017-0023

[B49] Rodríguez-Sánchez A. Salanova M. Cifre E. Schaufeli W. B. (2011). When good is good: a virtuous circle of self-efficacy and flow at work among teachers. *Rev. Psicol. Soc.* 26 427–441. 10.1174/021347411797361257

[B50] Rogala A. Cieślak R. (2019). Narzêdzie do pomiaru przekształcania pracy: właściwości psychometryczne polskiej wersji Job crafting scale. *Med. Pr.* 70 445–457. 10.13075/mp.5893.00822 31290486

[B51] Rowe G. Hirsh J. B. Anderson A. K. (2007). Positive affect increases the breadth of attentional selection. *Proc. Natl. Acad. Sci. U.S. A.* 104 383–388. 10.1073/pnas.0605198104 17182749 PMC1765470

[B52] Rudolph C. W. Katz I. M. Lavigne K. N. Zacher H. (2017). Job crafting: a meta-analysis of relationships with individual differences, job characteristics, and work outcomes. *J. Vocat. Behav.* 102 112–138. 10.1016/j.jvb.2017.05.008

[B53] Salanova M. Bakker A. B. Llorens S. (2006). Flow at work: evidence for an upward spiral of personal and organizational resources. *J. Happiness Stud.* 7 1–22. 10.1007/s10902-005-8854-8

[B54] Salanova M. Llorens S. Cifre E. MartÍNez I. M. Schaufeli W. B. (2003). Perceived collective efficacy, subjective well-being and task performance among electronic work groups: an experimental study. *Small Gr. Res.* 34 43–73. 10.1177/1046496402239577

[B55] Salanova M. Llorens S. Schaufeli W. B. (2011). Yes, I can, I feel good, and I JUST DO It!” On gain cycles and spirals of efficacy beliefs, affect, and engagement. *Appl. Psychol.* 60 255–285. 10.1111/j.1464-0597.2010.00435.x

[B56] Salanova M. Peiró J. M. Schaufeli W. B. (2002). Self-efficacy specificity and burnout among information technology workers: an extension of the job demand-control model. *Eur. J. Work Organ. Psychol.* 11 1–25. 10.1080/13594320143000735

[B57] Salanova M. Rodríguez-Sánchez A. M. Schaufeli W. B. Cifre E. (2014). Flowing together: a longitudinal study of collective efficacy and collective flow among workgroups. *J. Psychol.* 148 435–455. 10.1080/00223980.2013.806290 24946388

[B58] Sawyer R. K. (2003). *Group Creativity.Music, Theater, Collaboration.* Mahwah: Lawrence Erlbaum Associates.

[B59] Spector P. E. (2006). Method variance in organizational research: truth or urban legend? *Organ. Res. Methods* 9 221–232. 10.1177/1094428105284955

[B60] Stajkovic A. D. Lee D. Nyberg A. J. (2009). Collective efficacy, group potency, and group performance: meta-analyses of their relationships, and test of a mediation model. *J. Appl. Psychol.* 94 814–827. 10.1037/a0015659 19450017

[B61] Tims M. Bakker A. B. (2010). Job crafting: towards a new model of individual job redesign. *SA J. Ind. Psychol.* 36 1–9. 10.4102/sajip.v36i2.841

[B62] Tims M. Bakker A. B. Derks D. (2012). Development and validation of the job crafting scale. *J. Vocat. Behav.* 80 173–186. 10.1016/j.jvb.2011.05.009

[B63] Tims M. Bakker A. B. Derks D. (2013). The impact of job crafting on job demands, job resources, and well-being. *J. Occup. Health Psychol.* 18 230–240. 10.1037/a0032141 23506549

[B64] Tims M. Bakker A. B. Derks D. (2014a). Daily job crafting and the self-efficacy - performance relationship. *J. Manag. Psychol.* 29 490–507. 10.1108/JMP-05-2012-0148

[B65] Tims M. Bakker A. B. Derks D. (2014b). Job crafting and job performance: a longitudinal study. *Eur. J. Work Organ. Psychol.* 24 914–928. 10.1080/1359432X.2014.969245

[B66] Tims M. Derks D. Bakker A. B. (2016). Job crafting and its relationships with person-job fit and meaningfulness: a three-wave study. *J. Vocat. Behav.* 92 44–53. 10.1016/j.jvb.2015.11.007

[B67] Usher E. L. Pajares F. (2008). Sources of self-efficacy in school: critical review of the literature and future directions. *Rev. Educ. Res.* 78 751–796. 10.3102/0034654308321456

[B68] Van Katwyk P. T. Fox S. Spector P. E. Kelloway E. K. (2000). Using the Job-related affective well-being scale (JAWS) to investigate affective responses to work stressors. *J. Occup. Health Psychol.* 5 219–230. 10.1037/1076-8998.5.2.219 10784286

[B69] van Wingerden J. Niks I. M. W. (2017). Construction and validation of the perceived opportunity to craft scale. *Front. Psychol.* 8:573. 10.3389/fpsyg.2017.00573 28446893 PMC5388759

[B70] Weiss H. M. Cropanzano R. (1996). Affective events theory: a theoretical discussion of the structure, causes and consequences of affective experiences at work. *Res. Organ. Behav* 18 1–74.

[B71] Wood R. Bandura A. (1989). Social cognitive theory of organizational management university of new south wales. *Acad. Manag. Rev.* 14 361–384. 10.5465/AMR.1989.4279067

[B72] Wrzesniewski A. Dutton J. E. (2001). Crafting a job: revisioning employees as active crafters of their work. *Acad. Manag. Rev.* 26 179–201. 10.2307/259118

[B73] Xanthopoulou D. Bakker A. B. Demerouti E. Schaufeli W. B. (2012). A diary study on the happy worker: how job resources relate to positive emotions and personal resources. *Eur. J. Work Organ. Psychol.* 21 489–517. 10.1080/1359432X.2011.584386

